# Spinal Cord Toxoplasmosis: Mapping the Journey of a Rare Entity Through a Case Report and Review of the Literature

**DOI:** 10.3390/microorganisms14030535

**Published:** 2026-02-26

**Authors:** Sara Kamel Rey, Hessameldin Iranmanesh, Maya Hites, Sophie Elands, Sophie Henrard

**Affiliations:** 1 Clinic of Infectious Diseases, Hôpital Universitaire de Bruxelles (HUB), 1070 Brussels, Belgium; maya.hites@hubruxelles.be; 2 Neurology Department, Hôpital Universitaire de Bruxelles (HUB), 1070 Brussels, Belgium; hessamiranmanesh@gmail.com (H.I.); sophie.elands@hubruxelles.be (S.E.); 3 HIV Reference Center, Internal Medicine Department, Hôpital Universitaire de Bruxelles (HUB), 1070 Brussels, Belgium; sophie.henrard@hubruxelles.be

**Keywords:** HIV/AIDS, immunocompromised, spinal cord toxoplasmosis, *Toxoplasma gondii*

## Abstract

Toxoplasmosis remains the most frequent cause of cerebral lesions in patients with acquired immunodeficiency syndrome (AIDS), especially in those not receiving prophylaxis. Medullary involvement, although rare, can cause irreversible neurological damage. When associated with fever in the returning traveler, the etiological diagnosis of spinal cord lesions can be challenging due to the wide range of diagnostic possibilities. We report a unique case of spinal cord toxoplasmosis associated with *Salmonella* non-typhi bacteremia after a trip to Cameroon, revealing an advanced human immunodeficiency virus (HIV) infection in an otherwise healthy adult male. We also conducted a comprehensive review of reported spinal cord toxoplasmosis cases between the years 2000 and 2025 in both immunocompromised and immunocompetent patients. In our review, paraparesis, sensory loss, and urinary retention were the most frequent clinical presentations (52.17%; 56.52% and 47.84%, respectively), and the majority of the patients had concomitant cerebral lesions (78.26%). Diagnosis remains a challenge, with 48.0% of the reported cases diagnosed through histological detection of the parasite in central nervous system (CNS) tissue. Sulfadiazine–pyrimethamine with additional folinic acid and trimethoprim-sulfamethoxazole (TMP-SMX) remains the treatment of choice for treating cerebral toxoplasmosis in people living with HIV (PLHIV), with no particular recommendation regarding patients with spinal cord involvement. In the reviewed cases, neurological sequelae occurred in 52.2% of patients, and mortality was as high as 30.4%.

## 1. Introduction

Spinal cord lesions are rare but serious clinical entities that can cause irreversible neurological damage. When associated with fever in the returning traveler, the infectious hypothesis is the first one to consider, but even in this clinical scenario, the diagnosis can be challenging due to the wide range of diagnostic possibilities.

The distribution and imaging characteristics of the lesions, as well as the immune profile of the patient, frequently guide the clinician and help to differentiate between bacterial, fungal, parasitic, or viral etiologies. Spinal cord abscesses can be caused by Gram-positive or Gram-negative bacteria [1]; *Staphylococcus aureus* is the most prevalent pathogen in bacterial infections, followed by *Escherichia coli* and *Streptococcus* spp. [2,3]. Several cases of *Candida* spp. or *Aspergillus* abscesses have been reported [4,5], as well as spinal cord lesions caused by *Mycobacterium tuberculosis* and non-tuberculous mycobacteria [6], especially in immunocompromised hosts. Viral infections might present in the form of acute viral myelitis or as a chronic, progressive myelopathy. Enterovirus, poliovirus, and flaviviruses such as West Nile virus can be responsible for these lesions in the acute setting [7,8] and human T-lymphotropic virus-1 (HTLV-1) and human immunodeficiency virus (HIV) for more chronic presentations [9,10]. Parasitic infections, such as neurocysticercosis, toxoplasmosis, or schistosomosis, are rare causes of spinal cord lesions but must always be considered, particularly within the appropriate epidemiological context [11,12].

The risk for returned travelers to acquire specific infections varies according to the destination, setting, and activities undertaken, but in those returning from the tropics, malaria must always be excluded, and HIV must be considered in all settings [13].

Toxoplasmosis is the most common cause of cerebral mass lesions in patients with acquired immunodeficiency syndrome (AIDS) who are not receiving prophylaxis [14].

Typical manifestations of *T. gondii* encephalitis are focal ring-enhancing lesions with perilesional edema mainly in the brain, although cases with isolated spinal cord involvement have also been described in people living with HIV (PLHIV), immunosuppressed non-HIV patients, as well as in immunocompetent patients.

Beyond clinical manifestations, host cell invasion of *T. gondii* involves parasite factors linked to sialic acid-dependent interactions and chaperonin-related processes, highlighting the biological complexity underlying tissue tropism [15,16]. These mechanistic insights also motivate continued exploration of preventive and therapeutic strategies against toxoplasmosis [17,18].

Here we present a unique case of cerebral and spinal cord toxoplasmosis associated with *Salmonella* non-typhi bacteremia after a trip to Cameroon, revealing an advanced HIV infection in an otherwise healthy adult male. Data is scarce regarding spinal cord toxoplasmosis, especially in the returning traveler with HIV, where the diagnostic options for spinal cord lesions are even broader. Reviews on spinal cord toxoplasmosis have been conducted, the most recent of which includes data up until 2014 in patients with HIV [19]. We aim to update previous reviews in both immunocompetent and immunocompromised adult patients over the last 25 years (from 2000 to 2025). A comprehensive literature search was conducted across four electronic databases (PubMed, Google Scholar, Science Direct, and SciELO). Conference abstracts were included. All of the searches were conducted between 12 February 2025 and 13 June 2025. Almost all the cases included were published in English, except for one article in French and one in Spanish. Adult patients of any age were included if the diagnosis was confirmed by molecular identification of the parasite, histological evidence (including autopsy findings), or by a combination of radiological findings, clinical context, and positive serology. The combined terms used for the search were: (“spinal cord” OR “medullar” OR “transverse myelitis” OR “myelopathy”) AND (“toxoplasmosis”) AND (“immunocompetent” OR “immunocompromised”). To our knowledge, this is the first review to include cases of spinal cord toxoplasmosis in both immunocompetent and immunocompromised patients.

## 2. Case Presentation

A 32-year-old Cameroonian male living in Belgium was admitted to the emergency room for fever, abdominal, and lumbar pain 24 h after returning from a three-week trip to Cameroon. Laboratory investigations included blood tests, blood cultures, and a thick blood smear for malaria. The patient reported a history of malaria treated with intravenous (IV) artesunate during his stay in Cameroon. Twenty-four hours after admission, blood cultures were positive for *Salmonella enteritidis* and, therefore, treatment with ceftriaxone IV 2 g twice daily was initiated.

Neurological symptoms developed abruptly the next morning, when the patient experienced sudden bilateral leg weakness and was unable to stand up from a seated position without assistance. Within hours, he became paraparetic and required a wheelchair. There were no reported sensory symptoms initially, although urinary incontinence was observed.

Initial neurological examination revealed a bradyphrenic and mildly anosognosic patient with preserved cranial nerve function, except for a mild right lower facial paresis. There was no dysarthria or dysphagia. Muscle strength was preserved in the upper limbs (4/5), while lower limb strength was markedly reduced (right 2/5, left 1/5). Reflexes were present in the upper limbs and absent in the lower limbs. Plantar reflexes were flexor bilaterally. No sensory level was found, and vibratory and protopathic sensations were intact. Romberg testing was not possible due to the patient’s inability to stand or walk.

**Figure 1:**
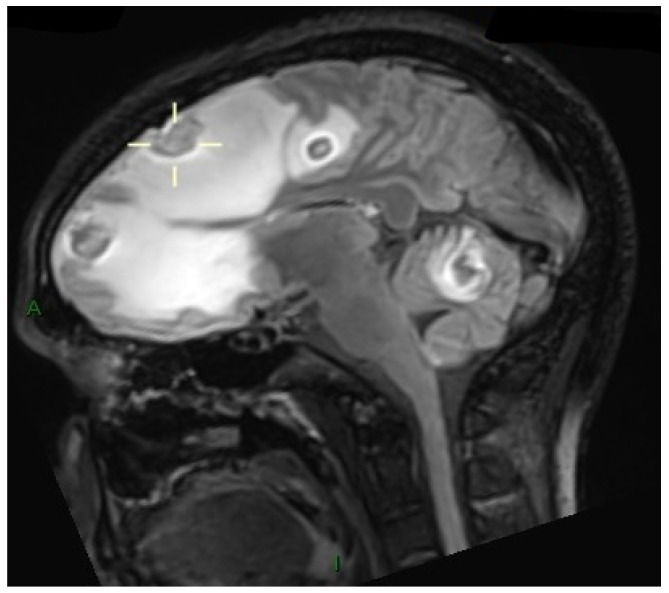
Fluid attenuated inversion recovery (FLAIR) magnetic resonance imaging (MRI) shows multiple supratentorial intraparenchymal enhancing lesions, predominantly located in the cortico-subcortical regions. Adjacent vasogenic edema is present around the largest lesions.

**Figure 2:**
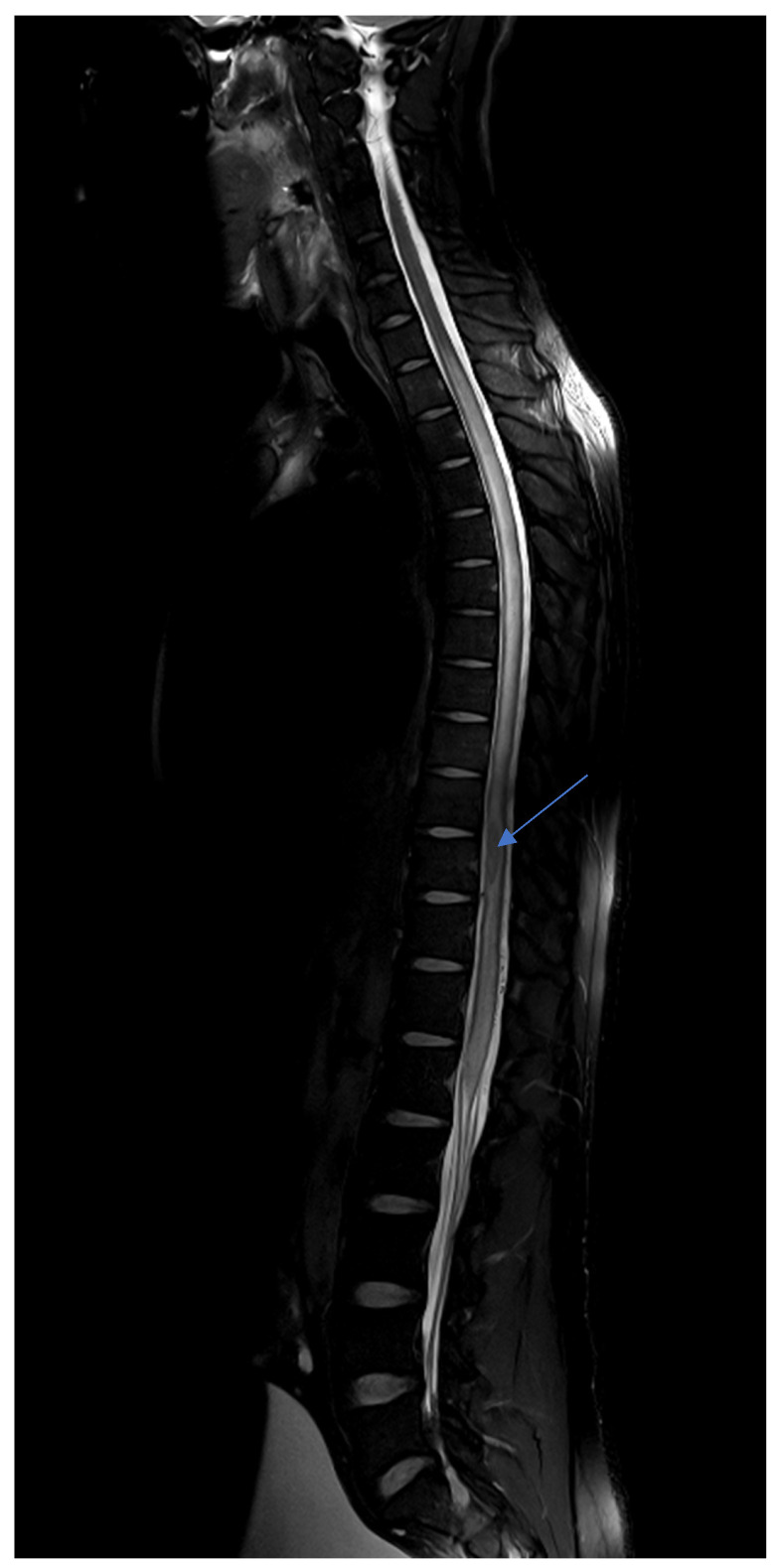
T2-weighted MRI shows an anterior intramedullary abscess at the level of the inferior border of the D9 vertebral body and the D10 vertebral body. A central intramedullary T2 hyperintense signal abnormality is observed, extending from the inferior aspect of C7 to the conus medullaris, associated with a mild to moderate diffuse enlargement of the spinal cord over the affected segment.

A magnetic resonance imaging (MRI) was immediately performed, showing multiple supratentorial and infratentorial ring-enhancing lesions with surrounding edema and mass effect (Figure 1), particularly in the left hemisphere with midline shift. In the spinal cord, an intramedullary abscess at D9-D10 with extensive surrounding T2 hyperintensity was noted, consistent with central cord edema (Figure 2). The cord was swollen from C7 to the conus, mimicking transverse myelitis.

HIV testing with enzyme-linked immunosorbent assay (ELISA) was reactive for HIV-1 and confirmed by a positive Western blot (WB) assay. His CD4+ lymphocyte count was 13 cells/mm^3^, and the viral load was 361,447 copies/milliliter (mL).

A brain biopsy was performed 12 h after the results of the MRI due to the possibility of different diagnostic aetiologies and the confounding factor of the *Salmonella* bacteriemia with a possible central nervous system (CNS) abscess. Immediately following the brain biopsy, treatment with sulfadiazine (3000 mg po (per os) twice daily) and pyrimethamine (75 mg once daily po after a 200 mg loading dose) was started and continued for two weeks. Folinic acid was supplemented as recommended [20]. Concomitantly, IV dexamethasone for the brain edema was prescribed and maintained for 10 days.

Serological tests revealed positive titers of Immunoglobulin G (IgG) for *T. gondii.* Histopathological results of the brain biopsy showed non-specific necrosis of brain parenchyma with no evidence of malignant cells and no findings compatible with mycobacteria, fungal, or parasitic infection. The Gram and acid-fast stains, as well as aerobic, anaerobic, and mycobacterial cultures of the brain biopsy, were negative. The polymerase chain reaction (PCR) for *Mycobacterium tuberculosis* was negative, and for *Toxoplasma gondii* was positive on the brain biopsy. No lumbar puncture was performed due to brain edema and mass effect.

Two weeks after the initiation of sulfadiazine–pyrimethamine, to both simplify the treatment and continue the antibiotic therapy for the *Salmonella* spp. bacteremia, the treatment was switched to trimethoprim-sulfamethoxazole (TMP-SMX) at the recommended dose (5 mg/kg of TMP and 25 mg/kg of SMX twice daily) to complete 6 weeks of therapy [20,21,22]. The strain was susceptible to all antibiotics tested except for fluoroquinolones. Blood cultures were monitored and remained negative.

To exclude further complications related to the *Salmonella* spp. bacteremia, a positron emission tomography and computed tomography (PET-CT) was performed. No signs of mycotic aneurysms, other infectious foci, or endocarditis were found.

Secondary prophylaxis with TMP-SMX 160/800 mg twice daily was continued after the 6-week treatment and stopped at 6 months, when an undetectable viral load was achieved, and the CD4 count was >200 cells/mm^3^.

At the three-month evaluation, the patient demonstrated a favorable neurological recovery. Motor function was preserved in all four limbs, with the exception of a mild distal weakness in the right lower limb and reduced pain and temperature sensation. Deep tendon reflexes were brisk in both lower limbs, with extension of reflexogenic zones, but without signs of spasticity or clonus. The patient also reported effort-related urinary incontinence. He was able to walk by himself with the aid of a single crutch. These findings are consistent with residual upper motor neuron involvement, predominantly affecting the right lower extremity, and reflect a partial yet encouraging recovery following targeted antimicrobial therapy.

**Figure 3:**
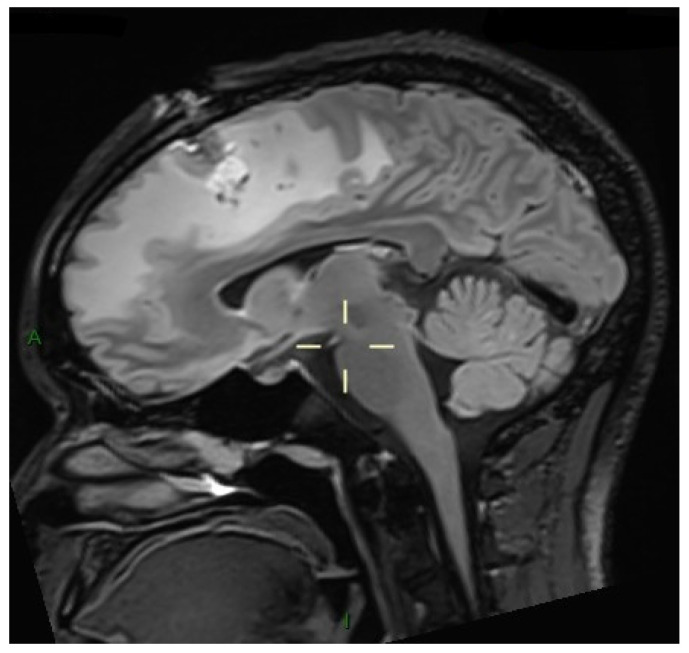
Axial MRI showing supratentorial ring-enhancing lesions with lobulated contours and surrounding vasogenic edema. Follow-up imaging demonstrates a marked reduction in lesion size, perilesional edema, and mass effect. The left frontal biopsy trajectory is also visible.

Follow-up MRI showed marked regression of both cerebral and spinal cord lesions and surrounding edema, reflecting optimal response to the treatment (Figure 3). The previously described ring-enhancing intramedullary lesion centered at the D9–D10 vertebral level remained stable in size. However, there was a marked reduction in the surrounding T2 hyperintense signal, indicating significant regression of the spinal cord edema initially observed.

## 3. Discussion

### 3.1. Results

The spinal cord remains a rare localization for *T. gondii* infection. Several cases have been reported since 1975, when the first case was described [23].

We reviewed the literature available on spinal cord toxoplasmosis in adult patients between the years 2000 and 2025 and found 25 cases (Table 1).

Twenty-three (92.0%) cases were reported in immunocompromised patients, and 2 (8.0%) in patients where there was no proven immunosuppression. Seventeen (68.0%) patients were seropositive for HIV, and amongst them, 13 were AIDS cases, and 3 others had unknown CD4+ cell count. Among the other 6 immunocompromised patients, 5 were diagnosed with malignant hemopathy. Among the HIV population, 11 patients (64.7%) were diagnosed with HIV at the time of the neurological presentation, with a median CD4 count of 58 cells/mm^3^ (min 10–max 270).

Paraparesis was present in 12/23 (52.17%) of patients. However, all patients presented limb weakness. Sensory loss (in 13/23; 56.52%), urinary retention (in 11/23; 47.84%), and altered reflexes (in 9/23; 39.13%) were the most frequent clinical presentations, and the thoracic spine was the most affected part of the spinal cord. Concomitant cerebral lesions were documented in 18/23 (78.26%) of patients.

Among the patients with available toxoplasma serology (18/25, 72.0%), only two (11.1%) had negative IgG titers.

Histological detection of the parasite in brain tissue (either tachyzoites with or without parasitic cysts/bradyzoites) yielded the diagnosis in almost half of the patients (12/25; 48.0%), and a positive PCR in the cerebrospinal fluid (CSF) yielded the diagnosis in almost one third of patients (7/25; 28.0%). In the remaining cases (5/25; 20.0%), the diagnosis was made based on a combination of clinical features, serology, imaging, and response to targeted therapy.

Different treatment regimens were administered, the sulfadiazine–pyrimethamine combination being the most frequent (10/16; 62.5%). Only 6 patients received corticosteroids. Four of the 25 patients (16.0%) received no treatment due to post-mortem diagnoses, and for five other patients, the information about the treatment was not available. In terms of outcomes, 4/23 (17.4%) patients fully recovered, 12/23 (52.2%) patients had neurological sequelae, and 7/23 (30.4%) died.

### 3.2. Review

The incidence of toxoplasmosis is higher in warm regions and increases with age. The seroprevalence is estimated to be 0.5–87.7% globally and of 32.1% in the European region [24,25] with a rate of 5.51 cases of congenital toxoplasmosis per 100,000 live births notified in 2021 [26]. In PLHIV, the risk of CNS toxoplasmosis largely reflects two determinants: local *T. gondii* IgG seroprevalence (reactivation reservoir) and the proportion of patients reaching advanced immunosuppression without prophylaxis and/or effective combination antiretroviral therapy (cART). Accordingly, toxoplasmic encephalitis (TE) incidence has markedly decreased in high-income settings after cART implementation (e.g., from 40.2 to 3.4 per 1000 person-years in a European national cohort) [27], whereas in sub-Saharan Africa—where HIV prevalence is higher, and *T. gondii* exposure among PLHIV is often substantial—neurotoxoplasmosis remains a frequent cause of focal CNS disease (e.g., 14.4% of HIV admissions in a Cameroonian tertiary hospital) [28]. Although spinal cord involvement is rare, it is reported predominantly in these same high-risk contexts, making the epidemiological setting a meaningful driver of pre-test probability for spinal cord disease.

*T. gondii* rarely causes disease in immunocompetent patients. Around 10–20% of cases will present with isolated cervical or occipital lymphadenopathy or may resemble a flu-like illness that will resolve after four to six weeks [14,22,29]. Although primary infections are usually asymptomatic in immunocompetent patients, a series of 44 cases of severe acquired toxoplasmosis in immunocompetent adults was described between 1998 and 2006 in French Guiana and attributed to higher virulent strains circulating in a forest-based cycle (in contrast to a domestic environment) [30]. No specific involvement of the CNS was described in any of the 44 cases. In the literature we reviewed, there was no proven cause of immunosuppression in two of the cases.

Patients with immunosuppressive conditions are at higher risk of developing the disease mainly through reactivation, due to impaired T-cell response.

HIV patients and patients under corticosteroid treatment can fail to produce antibodies, so serology can be negative, and therefore, a negative serology does not exclude toxoplasma diagnosis [31,32,33]. Toxoplasma PCR in the CSF has high specificity (95–100%) but low sensitivity 50–70% and a negative predictive value of 71–92% [20,34,35].

In the case described, a lumbar puncture was not performed due to the brain edema and mass effect present at diagnosis. A brain biopsy was performed within 24 h of hospital admission, and confirmed the diagnosis through PCR. The decision to perform a brain biopsy rather than a spinal cord biopsy was taken due to the anatomical accessibility and technical expertise available at our center.

From a radiological standpoint, spinal cord toxoplasmosis typically presents as focal intramedullary enhancing lesion(s) with cord swelling and extensive T2 hyperintensity surrounding edema. These conventional MRI findings are not specific and overlap with key differentials in advanced HIV, particularly primary CNS lymphoma and mycobacterial infection (e.g., intramedullary tuberculoma/abscess), which can also cause enhanced cord lesions and expansion. Spinal cord toxoplasmosis is rare; therefore, robust sensitivity and specificity estimates for spinal MRI patterns are not available. Nevertheless, data from cerebral disease can help frame discriminative imaging signs. The “eccentric target sign” is highly specific but poorly sensitive for toxoplasmosis [36]. Diffusion-weighted imaging tends to show higher Apparent Diffusion Coefficient (ADC) in toxoplasmosis than in lymphoma (with high-specificity ADC thresholds described) [37], and a perfusion MRI often demonstrates lower relative Cerebral Blood Volume (rCBV) in toxoplasmosis compared with lymphoma [38].

In the specific context of HIV infection, especially when CD4 count is <200 cells/mm^3^, a presumptive diagnosis of TE can be made through a combination of a positive toxoplasma serology, typical neuroradiology imaging, a compatible clinical setting, and response to therapy, as lumbar puncture might not always be feasible, and brain biopsy is not available and too invasive. Targeted therapy should be started without delay and imaging reassessed within 10–14 days, as well as neurological examination carefully monitored on a daily basis. If there is no radiological and/or clinical improvement, a brain biopsy should be performed to rule out alternative diagnoses such as primary CNS lymphoma, tuberculosis of the CNS, or bacterial abscesses [39]. The role of brain biopsy is crucial in clinical scenarios where imaging characteristics and/or laboratory or microbiological findings can suggest an alternative diagnosis, such as the case presented here. Morbidity and mortality rates reported after brain biopsy are non-negligible (7.5% to 12% and 0% to 3.1%, respectively), highlighting the importance of performing this technique in centers with experience [40,41,42].

Unlike the reviewed cases, where 48.0% of the cases were confirmed by histology, the parasite was not visualized in the brain biopsy specimen by the anatomical pathology service. In contrast to molecular methods, histopathology results remain dependent on the operator.

Sulfadiazine–pyrimethamine with additional folinic acid remains the treatment of choice for treating cerebral toxoplasmosis in PLHIV [20,43,44], with no particular recommendation regarding patients with spinal cord involvement. For those in settings with limited resources, without access to this medication, and in situations requiring IV treatment, when pill burden is high or when significant secondary effects occur, TMP-SMX is a viable alternative that has been shown to be effective and safe [20,45,46,47]. In our case, a successful treatment with sulfadiazine–pyrimethamine was started and later simplified to TMP-SMX.

Clindamycin-pyrimethamine remains a second choice in HIV patients, as two studies from the 90s proved a higher risk of progression and less survival compared to TMP-SMX [43,48]. However, clindamycin-pyrimethamine remains the main alternative to sulfadiazine–pyrimethamine in patients who develop toxoplasmosis after hematopoietic stem-cell transplantation [49].

In addition to established regimens, repurposing and screening efforts have identified lumefantrine as a candidate with reported anti-*T. gondii* activity in experimental settings, although clinical evidence for CNS disease remains limited [50,51].

There are no randomized clinical trials assessing the use of corticosteroids in the treatment of cerebral and spinal cord toxoplasmosis, and retrospective studies reported no benefit [28]. Nonetheless, when cerebral edema and/or lesions with mass effect are present, steroids are recommended, and should be discontinued as soon as clinically feasible to prevent secondary effects and other infectious complications, such as cytomegalovirus (CMV) or herpes reactivation in the specific context of immunocompromised patients [20,34].

In the cases we reviewed, different corticosteroid regimes were used. Doses significantly varied as well as duration. Information concerning the duration of therapy was lacking in most of the reviewed cases. However, based on our experience, a 6-week treatment duration seems reasonable for spinal cord lesions with adjunctive corticosteroid treatment to diminish surrounding edema. The optimal duration of corticosteroid treatment remains unknown.

Our review highlights that one out of two patients with spinal cord toxoplasmosis survived with neurological sequelae and one out of three patients died, reflecting outcomes similar to those reported in previous series [19,52] that included patients in the late 80s and 90s with more limited diagnostic methods, and when treatment of HIV infection was frequently toxic and inefficient. These results, in the era of cART and where the Joint United Nations Program on HIV and AIDS (UNAIDS) targets are 95-95-95 in 2030 (95% of all PLHIV should have a diagnosis, 95% of whom should be taking lifesaving antiretroviral treatment, and 95% of PLHIV on treatment should achieve a suppressed viral load), outline the importance of quickly identifying and treating rapidly rare presentations of an already infrequent disease.

Our case was particularly challenging due to the confounding factor of non-typhoidal *Salmonella* spp. bacteremia, responsible for up to 39% of community acquired blood stream infections in sub-Saharan Africa, with an average case fatality rate of 19% [53].

Non-typhoidal *Salmonella* spp. infections cause mainly gastrointestinal infections, although invasive forms with bacteremia and metastatic foci—particularly in immunocompromised patients—such as endocarditis, osteomyelitis, and CNS abscesses have been well documented.

Hirai et al. [54] published a case report and review of the literature of non-typhoidal *Salmonella* spinal epidural abscess, where symptoms at presentation were similar to those described in our patient. This highlights the importance of a thorough differential diagnosis of fever and CNS lesions in the returning traveler.

**Table 1:** Main clinical features, diagnosis, treatment, and outcomes of the reviewed cases.

Author, Year [Ref]	Gender/ Age (Years)	Immunosuppression/ Risk Factor for the Infection	If HIV, De Novo Diagnosis	Clinical Features	Spine Location	Concomitant Brain Involvement	Toxoplasma Serology (IgG)	Diagnostic Methods	Treatment	Outcomes
London, 2023 [55]	M/72	Allogenic stem cell transplantation for AML and hepatic GVHD treated with prednisone	N/A	Paraparesis, urinary retention, brisk lower limb reflexes	C1-T8	No	Negative	CSF-PCR ^1^	TMP-SMX for 6 weeks + prednisone at the same dose	Partial recovery. Permanent paraplegia and urinary retention)
Baeza, 2021 [56]	M/32	HIV (CD4 17 cells/mm^3^)	Yes	Paraparesis, perineal anesthesia, absence of reflexes, and acute urinary retention	Conus medullaris	Yes—frontal and insular lobes	Positive	CSF-PCR ^1^	Sulfadiazine–pyrimethamine	Improvement
Mohole, 2019 [57]	F/36	HIV (CD4 60 cells/mm^3^)	Yes	Sensory and motor deficit and left Achilles areflexia. Severe back pain	T6-L1	No	Positive	Histopathological study (pseudocysts) ^2^	Sulfadiazine–pyrimethamine for 6 weeks + laminectomy	Improvement of the back pain and increased movement in the lower extremities
Bocca, 2019 [58]	M/29	HIV (unknown CD4 count)	No	Left-hand weakness and numbness. Neck pain and urinary retention	C4-C6	Yes	Positive	Postmortem histopathological study (bradyzoites) ^2 3^	Not specified	Death
Martinot, 2019 [59]	M/31	None—boar meat consumption	N/A	Brown–Séquard syndrome	C3-C4	No	IgM and IgG positive	Seroconversion for *T. gondii* antibodies, exposition, clinical features and response to therapy	Sulfadiazine–pyrimethamine for 6 weeks	Complete resolution of symptoms
Mahendran, 2018 [60]	M/34	HIV (unknown CD4 count)	Yes	Paraparesis and urinary retention	C1-C2	Yes	Positive	Presumptive diagnosis ^4^	Sulfadiazine–pyrimethamine	Improvement
Sireesha, 2018 [61]	M/35	None	N/A	Paresthesia of the left upper limb	C2-C6	Yes, after administration of corticosteroids	Unknown	Postmortem histopathological study (bradyzoites and tachyzoites) ^2 3^	No treatment	Death
Pérez, 2017 [62]	F/33	HIV (CD4 11 cells/mm^3^)	No	Paraparesis, urinary retention, and sensory level	T8	Yes	Positive	Presumptive diagnosis ^4^	TMP-SMX + 7 days of corticosteroids	Partial recovery. Residual urinary retention
Sbeih, 2016 [63]	M/40	HIV (unknown CD4 count)	Yes	Tetraparesis, hypoesthesia, urinary retention, chronic constipation, and abdominal pain	C1-T4	Yes—both cerebral hemispheres	Positive	Histopathological study (bradyzoites and tachyzoites) ^2^	TMP-SMX and pyrimethamine for 8 weeks + laminectomy	Complete resolution of lesions.
García, 2014 [19]	M/48	HIV (CD4 36 cells/mm^3^)	Yes	Right arm monoparesis, hypoesthesia, urinary retention, dysarthria, brisk upper limb reflexes, and back pain	C4-T10	Yes—both frontal hemispheres	Positive	Histopathological study (bradyzoites within cysts) ^2^	Sulfadiazine–pyrimethamine + corticosteroids	Partial recovery. Residual right-sided hypoesthesia
Krishnaswamy, 2014 [64]	Not described	Stem cell transplantation for CML	N/A	Not specified	Unknown	Yes—multiple lesions	Unknown	Postmortem histopathological study ^2 3^	No treatment	Death
Agrawal, 2014 [65]	M/40	HIV (CD4 94 cells/mm^3^)	Yes	Paraparesis and brisk reflexes, anesthesia, and urinary retention	T9	Yes—occipital lobe	Positive	CSF-PCR ^1^	TMP-SMX	Almost full recovery (Strength IV/V)
Rodríguez, 2013 [52]	M/40	HIV (CD4 60 cells/mm^3^)	No	Paraparesis, anesthesia, and urinary retention	T10-T12	Unknown	Positive	Histopathological study (tachyzoites) ^2^	TMP-SMX + clindamycin	Partial recovery. Right foot drop sequela
Sidani, 2013 [66]	F/54	HIV (CD4 22 cells/mm^3^)	Yes	Paraparesis and back pain	T5-T8	Yes—multiple lesions	Unknown	Histopathological study (tachyzoites and pseudocysts) ^2^	Medical treatment non-specified + laminectomy	Not specified
Denes, 2013 [67]	M/54	HIV (CD4 164 cells/mm^3^)	Yes	Monoparesis of the right arm	C4-T2	No	Unknown	PCR for *T. gondii* positive in spinal cord biopsy	Not specified	Partial recovery. Undefined residual symptoms
Maroski, 2013 [68]	F/41	HIV (CD4 10 cells/mm^3^)	Yes	Tetraparesis, urinary incontinence, altered mental status	Conus medullaris	Yes—multiple lesions	Unknown	CSF-PCR	Not specified	Not specified
Kung, 2011 [69]	M/34	HIV (CD4 67 cells/mm^3^)	No	Paraparesis, sensory level at L4, constipation, and abnormal reflexes	T11-T12	Yes	Negative	Histopathological study (cysts) ^2^	Sulfadiazine–pyrimethamine + laminectomy	Suspected IRIS and death
De Bonis, 2011 [70]	M/44	HIV (CD4 270 cells/mm^3^) and intense chemotherapy for Burkitt lymphoma	No	Paraparesis, perineal anesthesia, and absent Achilles reflexes	Conus medullaris	Yes—right frontal lobe	Positive	Histopathological study (tachyzoites) ^2^	Sulfadiazine–pyrimethamine. The disease developed under correct prophylactic treatment	Full recovery
García-Gubern, 2010 [32]	M/40	HIV (CD4 56 cells/mm^3^)	Yes	Paraparesis, anesthesia, and urinary retention	Cervical and at cauda equina	Yes—left frontal lobe and internal capsule	Positive	Presumptive diagnosis ^4^	sulfadiazine–pyrimethamine + corticosteroids	Incomplete resolution at 3 weeks of treatment
Herold, 2009 [71]	F/56	Chronic lymphocytic leukemia	N/A	Paresthesia and pain of the right arm, hyporeflexia	C5-C6	Yes	Unknown	CSF-PCR and postmortem histological study (tachyzoites and bradyzoites) ^2 3^	Sulfadiazine–pyrimethamine + clindamycin	Death
Arshad, 2009 [72]	F/46	HIV (CD4 60 cells/mm^3^)	No	Paraparesis and leg pain	Thoracic	Yes	Positive	Presumptive diagnosis ^4^	Sulfadiazine–pyrimethamine + corticosteroids for 8 weeks	Resolution of symptoms
Pittner, 2008 [73]	M/46	HIV (CD4 44 cells/mm^3^)	Yes	Monoparesis of the right lower limb, altered thermal sensitivity, urinary retention, lumbar pain	D12-L1	Yes	Positive	CSF-PCR	Clindamycin (substituted by atovaquone due to suspected allergy)—Pyrimethamine + corticosteroids	Partial recovery. Residual right paresis and walking difficulties
Straathof, 2001 [74]	M/60	Autologous stem cell transplantation for MM and vasculitis treated with corticosteroids	N/A	Brown–Séquard syndrome and abnormal gait	T7	No	Positive	CSF-PCR	Not specified. Corticosteroids were discontinued	Partial recovery. Residual dysesthesia and slight walking difficulties
Maciel, 2000 [75]	M/51	T cell leukemia-lymphoma HTLV-1 related	N/A	Paraplegia, hyperreflexia, and altered mental status	Not described	Yes—multiple lesions	Unknown	Postmortem histopathological study (bradyzoites and tachyzoites) ^2 3^	No treatment	Death
Nakane, 2000 [76]	F/49	T-cell leukemia-lymphoma treated by autologous stem cell transplantation	N/A	Not described	Not described	Unknown	Positive	Postmortem histopathological study (cysts) ^2 3^	No treatment	Death

AML (acute myeloid leukemia); CML (chronic myeloid leukemia); CSF (cerebrospinal fluid); F (female); GVHD (graft versus host disease); HIV (human immunodeficiency virus); HTLV-1 (human T-lymphotropic virus-1); IRIS (immune reconstitution inflammatory syndrome); M (male); MM (multiple myeloma); N/A (non-applicable); PCR (polymerase chain reaction); TMP-SMX (trimethoprim-sulfamethoxazole). ^1^ CSF-PCR refers to a positive PCR in the cerebrospinal fluid for *T. gondii*, establishing the diagnosis. ^2^ Histopathological studies refer to a sample taken through a spinal cord biopsy. ^3^ Postmortem diagnosis was established through autopsy. ^4^ Presumptive diagnosis was defined by the combination of appropriate clinical context, radiological findings, and positive *T. gondii* serology.

## 4. Conclusions

Spinal cord toxoplasmosis is a rare but severe disease almost exclusively observed in immunocompromised hosts. In PLHIV, specifically those that are not treated with antiretrovirals, toxoplasmosis should always be considered when medullary involvement is present, even when co-infections occur, as observed in this case report.

In spite of better immunovirological control of HIV infection and better HIV treatment coverage, cases of spinal cord toxoplasmosis continue to be reported. This can be explained by a higher number of stem cell transplantations and more intense chemotherapies for hematological malignancies, given that patients under these conditions can also develop medullary toxoplasmosis.

There are several limitations to our review. First, only a small number of cases were included due to the rarity of this entity. Second, several of the included articles lacked methodological rigor, resulting in missing key information. Third, the heterogeneity of the included cases—in terms of patient populations (HIV-positive, non-HIV immunosuppressed, and immunocompetent patients), as well as diagnostic and treatment approaches—limits direct comparisons.

In light of the high burden of neurological sequelae and mortality observed in reported cases, preventive strategies remain highly desirable. Experimental vaccine approaches targeting antigens such as surface antigen 1 (SAG1) and sialic acid–binding proteins have shown protective signals in animal models, warranting further translational work [17,18].

## Data Availability

The original contributions presented in this study are included in the article. Further inquiries can be directed to the corresponding author.
